# Lifestyle Modification and Bariatric Surgery in Type 2 Diabetes Remission: Short title: Diabetes Remission Strategies in T2DM

**DOI:** 10.31661/gmj.v15i.4153

**Published:** 2026-09-26

**Authors:** Noor AlFahhad

**Affiliations:** ^1^ Department of Family Medicine, College of Medicine, Qassim University, Buraidah, Saudia Arabia

**Keywords:** Type 2 Diabetes Mellitus, Bariatric Surgery, Diabetes Remission, Lifestyle Modification, Weight Loss

## Abstract

Type 2 diabetes mellitus (T2DM) has traditionally been regarded as a chronic and progressively worsening disease. This paradigm is now challenged by evidence showing that remission is achievable in selected patients, particularly when interventions produce substantial and sustained weight loss. This narrative review synthesizes current evidence on lifestyle modification and bariatric/metabolic surgery as remission-inducing strategies, with emphasis on mechanisms, comparative efficacy, predictors of response, and limitations. Intensive lifestyle interventions, especially very-low-calorie diets and total diet replacement programs, can induce normoglycemia in selected individuals through reductions in hepatic and pancreatic fat, improved insulin sensitivity, and partial recovery of β-cell function. However, durability is limited and depends strongly on sustained weight loss. Bariatric/metabolic surgery, particularly Roux-en-Y gastric bypass and sleeve gastrectomy, achieves higher and more durable remission rates than non-surgical approaches. These effects are mediated by both weight loss and weight-independent mechanisms, including enhanced incretin signaling, altered bile acid metabolism, and gut microbiota changes. Consistent predictors of remission include shorter diabetes duration, lower baseline HbA1c, reduced treatment burden, greater weight loss, and preserved β-cell function. Despite these advances, relapse remains common, long-term randomized data are limited, and important barriers persist, including procedural risk, cost, and inequitable access. Overall, the evidence supports a shift from a model of inevitable progression toward one of potentially reversible metabolic dysfunction, with important implications for early, remission-oriented care.

## Introduction

Type 2 diabetes mellitus (T2DM) is a leading global cause of morbidity, mortality, and healthcare expenditure, with prevalence projected to exceed 1.3 billion cases by 2050 [1, 2]. The economic burden is substantial, accounting for approximately 1.7-1.8% of global gross domestic product and continuing to rise [3]. Despite this scale, the dominant clinical paradigm has remained largely unchanged.

T2DM is typically managed as a chronic, progressive disease requiring stepwise intensification of pharmacotherapy [4]. Evidence from intensive dietary interventions and bariatric/metabolic surgery demonstrates that normoglycemia can be restored and β-cell function can partially recover in a substantial proportion of patients [5, 6, 7].

These findings challenge the assumption of irreversible β-cell failure and support a model in which T2DM reflects potentially reversible metabolic dysfunction, particularly in earlier disease stages [6, 7].

An international consensus defines remission as HbA1c <48 mmol/mol (6.5%) for at least 3 months without glucose-lowering medication, while emphasizing uncertainties regarding durability and long-term risk [8, 9]. Remission remains uncommon in routine clinical care, reflecting both biological constraints and implementation barriers [10]. Substantial weight loss is central to remission because it reduces ectopic fat, restores insulin sensitivity, and improves β-cell function [11]. Lifestyle intervention and bariatric/metabolic surgery both target these pathways, but they differ markedly in magnitude, durability, and scalability of effect [12, 13].

This review focuses on mechanisms, comparative efficacy, predictors of success, and key limitations, with the aim of clarifying how remission-oriented care can be integrated into contemporary diabetes management.

## Pathophysiology of T2DM

T2DM arises from the interaction between insulin resistance and progressive β-cell dysfunction, driven primarily by chronic energy excess and ectopic lipid deposition [4, 14].

### Insulin Resistance

Insulin resistance in the liver, skeletal muscle, and adipose tissue is an early and central abnormality. Hepatic insulin resistance impairs suppression of gluconeogenesis, increasing fasting glucose, while reduced peripheral glucose uptake contributes to postprandial hyperglycemia [4, 15]. A major mechanistic driver is ectopic lipid accumulation, particularly diacylglycerols and ceramides, which disrupt intracellular insulin signaling pathways. This indicates that metabolic dysfunction is driven less by total adiposity than by lipid distribution and organ-specific fat deposition [16, 17].

β-Cell Dysfunction Progression to overt diabetes occurs when pancreatic β-cells can no longer compensate for insulin resistance. This failure is mediated by chronic exposure to elevated glucose and fatty acids, leading to glucotoxicity, lipotoxicity, oxidative stress, and endoplasmic reticulum stress [15, 17]. Ectopic pancreatic fat further impairs insulin secretion and is strongly associated with diabetes risk, supporting a lipotoxic contribution to β-cell dysfunction [16, 18].

Ectopic fat and the twin‑cycle hypothesis The twin-cycle hypothesis provides a unifying framework linking hepatic and pancreatic fat accumulation to T2DM pathogenesis [17, 18]. Excess caloric intake promotes hepatic fat accumulation, driving insulin resistance and increased hepatic glucose output. Compensatory hyperinsulinemia further enhances hepatic lipogenesis, creating a self-reinforcing cycle [19, 20]. In parallel, lipid accumulation in the pancreas impairs β-cell function, ultimately leading to hyperglycemia and clinical diabetes [17, 21].

## Definitions and Criteria for Diabetes Remission

Definitions of T2DM remission have evolved substantially, reflecting a shift from complex, multi-tiered classifications toward a pragmatic and clinically applicable framework [9, 22]. Earlier consensus statements distinguished between partial, complete, and prolonged remission using different glycemic thresholds and duration criteria [22]. Although conceptually rigorous, this approach was difficult to apply consistently across clinical and research settings [8].

The current international consensus defines remission as HbA1c <48 mmol/mol (<6.5%) for at least 3 months in the absence of glucose-lowering therapy [8, 9, 22]. This definition prioritizes feasibility and standardization, facilitating comparison across studies and implementation in routine care [22].

The current definition does not capture heterogeneity in underlying disease biology, nor does it account for relapse risk or long-term metabolic health [8]. In particular, reliance on a single glycemic threshold may obscure clinically meaningful differences between transient glycemic improvement and durable disease modification [8, 9].

Furthermore, variability in definitions across earlier studies continues to complicate interpretation of remission rates and long-term outcomes [9]. This heterogeneity limits comparability between interventions and may lead to overestimation of durable remission [8].

## Lifestyle Interventions for T2DM Remission

Lifestyle intervention is a primary strategy for achieving T2DM remission, with the magnitude and durability of weight loss emerging as the dominant determinants of success. However, outcomes vary substantially according to intervention intensity, adherence, and long-term maintenance [23].

### Weight Loss Interventions

Clinically meaningful weight reduction, typically ≥10-15 kg, is the most consistent predictor of remission, primarily through reduction of hepatic and pancreatic fat, restoration of insulin sensitivity, and recovery of β-cell function [11, 24]. The DiRECT trial established a clear dose-response relationship between weight loss and remission [25]. A structured primary care-led program incorporating total diet replacement achieved remission in 46% of participants at 12 months and 36% at 2 years, with substantially higher rates among those maintaining ≥10 kg weight loss [24, 25]. Longer-term follow-up demonstrates attenuation over time but sustained benefit relative to usual care [26]. Replication in real-world settings, including DiRECT-Australia and the NHS Path to Remission program, confirms the effectiveness of total diet replacement-based interventions, although lower remission rates reflect reduced adherence and implementation variability [27, 28]. These findings reinforce weight loss as the principal mechanism underlying remission while highlighting the challenge of long-term maintenance [28, 29].

Dietary Strategies: Efficacy and Limitations A range of dietary approaches can induce weight loss and improve glycemic control, but differences in remission outcomes are driven largely by adherence and energy deficit rather than macronutrient composition [30]. Low-carbohydrate diets (<130 g/day) improve short-term remission rates and metabolic outcomes compared with higher-carbohydrate diets, although these effects diminish over time and are highly adherence-dependent. Very-low-carbohydrate and ketogenic diets do not reproducibly confer additional benefit when adherence is considered [31].

### Mediterranean Diet

Mediterranean-style diets, characterized by high intake of unsaturated fats, fiber, and polyphenols, produce more modest weight loss but confer sustained improvements in glycemic control and cardiovascular risk [11, 32]. Randomized data suggest higher remission rates compared with low-fat diets, although the certainty of evidence remains limited [33]. Intermittent energy restriction, including time-restricted eating and alternate-day fasting, has shown comparable weight loss and remission rates to continuous energy restriction in some studies; however, evidence remains heterogeneous and long-term sustainability is uncertain [28].

Physical Activity: Supportive but Insufficient Alone Physical activity improves insulin sensitivity independently of weight loss by enhancing skeletal muscle glucose uptake and insulin signaling [34]. Both aerobic and resistance training reduce HbA1c and improve body composition, with combined modalities producing the greatest metabolic benefit [35]. Exercise also reduces visceral adiposity, a major driver of insulin resistance, even without marked weight reduction [34, 36]. Nevertheless, physical activity alone rarely induces remission because it typically does not achieve the degree of weight loss required [36]. Its major contributions are enhancement of weight loss, preservation of lean mass, and improvement of long-term metabolic health when combined with dietary interventions [37].

## Bariatric (Metabolic) Surgery and Diabetes Remission

Bariatric surgery remains the most effective intervention currently available for achieving and sustaining remission of T2DM, consistently outperforming lifestyle and medical therapy in both magnitude and durability of glycemic improvement [7, 13].

**Table:** Table1. Bariatric Procedures and Diabetes Remission Outcomes

Procedure	Mechanism	Typical Weight Loss	Remission Rate	Key Advantages	Limitations
Roux-en-Y gastric bypass [44, 45, 46, 47]	Restrictive + hormonal	25-35%	60-80%	High efficacy, durable remission	Surgical complexity, micronutrient deficiency
Sleeve gastrectomy [44, 45, 48, 49]	Restrictive + hormonal	20-30%	50-70%	Widely used, lower risk than RYGB	Slightly lower remission durability
Biliopancreatic diversion with duodenal switch [50, 51, 52]	Restrictive + hormonal	30-40%	70-90%	Highest remission rates	High complication risk
Adjustable gastric band [53, 54]	Restrictive	15-20%	30-45%	Less invasive	Lowest efficacy, declining use

Mechanisms: Beyond Weight Loss Although substantial and sustained weight loss is a major driver of remission, bariatric surgery exerts additional weight-independent metabolic effects that contribute to its superior efficacy [38]. Procedures such as Roux-en-Y gastric bypass and sleeve gastrectomy induce rapid postprandial increases in glucagon-like peptide-1 (GLP-1), enhancing glucose-dependent insulin secretion, suppressing glucagon, delaying gastric emptying, and reducing appetite [38, 39]. These changes contribute to early improvements in glycemia, often preceding significant weight loss [38, 40]. Surgery also alters bile acid metabolism and gut microbiota composition, activating signaling pathways such as FXR and TGR5 that improve insulin sensitivity and energy homeostasis [41, 42]. These mechanisms interact to create a metabolically favorable state that supports sustained glycemic control. In parallel, early caloric restriction and rapid reductions in hepatic fat lead to prompt improvements in hepatic insulin sensitivity, contributing to early normalization of fasting glucose [40].

### Comparative Effectiveness

Remission rates vary by procedure, reflecting differences in anatomical and physiological effects (Table1). Randomized controlled trials consistently demonstrate the superiority of bariatric surgery over non-surgical interventions. In a landmark trial, remission at 3 years was achieved in 40% of patients undergoing Roux-en-Y gastric bypass and 29% following gastric banding, compared with no remission in the intensive lifestyle group [12]. At 5 years, remission persisted in 30% of surgical participants, while remaining negligible in non-surgical controls [13]. Notably, surgical benefit extends beyond traditional eligibility thresholds, with improved glycemic outcomes observed even in individuals with BMI <35 kg/m² [43].

### Durability and Relapse

Despite high initial remission rates, relapse is common over time. Remission typically peaks within 1-2 years and declines thereafter, particularly in patients with longer diabetes duration or suboptimal weight loss [55, 56]. Relapse should not be interpreted as treatment failure. Even after recurrence of hyperglycemia, patients generally maintain improved glycemic control, reduced medication requirements, and lower cardiometabolic risk compared with pre-intervention states. This supports the conceptualization of bariatric surgery as a disease-modifying intervention rather than a definitive cure [47, 57].

## Bariatric Surgery vs Lifestyle Intervention for Diabetes Remission

Direct comparisons reproducibly demonstrate the superior efficacy of bariatric surgery over lifestyle intervention in achieving T2DM remission [13]. Randomized controlled trials show markedly higher remission rates after surgical procedures, with sustained benefits extending beyond 5 years, whereas remission in lifestyle intervention groups is typically modest and less durable [12, 13]. Network meta-analyses estimate that bariatric surgery markedly increases the likelihood of remission compared with non-surgical approaches [58, 59]. These large relative effects should be interpreted cautiously because they are partly driven by low remission rates in control groups; nevertheless, even conservative estimates confirm a substantial absolute benefit of surgery [43, 60, 61].

### Magnitude and Durability of Effect

The main distinction between interventions lies not only in remission rates, but in the magnitude and sustainability of metabolic change. Bariatric surgery reliably induces greater and more sustained weight loss, which underpins its superior remission outcomes [50]. In contrast, intensive lifestyle interventions can achieve comparable short-term remission rates under controlled conditions, particularly when large magnitude weight loss is achieved [27]. By comparison, these effects are highly dependent on adherence and typically attenuate over time due to weight regain [24, 62]. Clinically, lifestyle approaches depend on adherence, whereas surgery generates physiological changes that help sustain metabolic outcomes [13].

### Mechanistic Distinction

Although both approaches converge on weight loss as a central mechanism, bariatric surgery exerts additional weight-independent effects that are not replicated by lifestyle intervention alone [63]. These include enhanced incretin signalling, altered bile acid metabolism, and changes in gut microbiota, which collectively contribute to improved glucose homeostasis [38, 41]. As a result, surgical intervention often produces more rapid and pronounced glycemic improvements, including early normalisation of fasting glucose prior to substantial weight loss [5].

### Patient Selection and Clinical Context

The relative effectiveness of each approach is strongly modified by patient characteristics. Lifestyle interventions are most effective in individuals with shorter diabetes duration, lower baseline HbA1c, and preserved β-cell function profiles associated with greater metabolic reversibility [23]. In contrast, bariatric surgery remains effective across a broader spectrum of disease severity, including patients with longer-standing diabetes or higher treatment burden, although remission rates decline with advancing disease duration [64]. These differences highlight the importance of individualized treatment selection, rather than viewing interventions as competing alternatives [65].

### Scalability, Risk, and Implementation

Despite its superior efficacy, bariatric surgery is limited by cost, resource requirements, procedural risk, and restricted access [50]. In contrast, lifestyle interventions are scalable and broadly applicable but constrained by adherence and long-term effectiveness. Thus, the choice between interventions reflects a trade-off between efficacy and scalability, with surgery offering greater metabolic impact at the cost of invasiveness and accessibility [13].

## Predictors of Diabetes Remission

**Table:** Table2. Major Predictors of T2DM Remission Across Lifestyle and Surgical Interventions

Predictor	Favorable profile	Mechanistic interpretation	Clinical implication
Duration of T2DM [21, 36]	Short duration, ideally <5-6 years	Greater preservation of β-cell function and metabolic reversibility	Supports earlier intervention
Baseline HbA1c [68, 70]	Lower HbA1c at baseline	indicates less severe dysglycemia and lower metabolic stress	Higher likelihood of remission
Treatment burden [21, 66, 70]	No insulin or lower medication burden	Proxy for preserved β-cell reserve and less advanced disease	Useful pragmatic predictor in routine care
Magnitude of weight loss [57, 64]	Greater weight loss, especially ≥10-15%	Reduces hepatic and pancreatic fat, improves insulin sensitivity	Strongest modifiable determinant
β-cell reserve [36, 68, 72]	Higher C-peptide / preserved insulin secretion	Enables maintenance of normoglycemia after intervention	Critical for durable remission

Across both lifestyle and surgical interventions, remission is not random. A consistent set of clinical predictors determines the likelihood of success, largely reflecting the underlying stage of disease and the degree of residual metabolic reversibility [66, 67]. Table2 presented the major predictors of T2DM remission across lifestyle and surgical interventions

### Duration of Diabetes

Diabetes duration is one of the strongest and most reproducible predictors of remission. Shorter disease duration is consistently associated across studies with higher remission rates [10, 21, 68]. This association is biologically plausible: earlier disease is more likely to preserve β-cell function and therefore retain the capacity for metabolic recovery. In contrast, prolonged disease duration likely reflects more advanced and less reversible β-cell failure [56, 69].

### Baseline Glycaemia and Treatment Burden

Lower baseline HbA1c and reduced treatment intensity, particularly the absence of insulin therapy, are also strong predictors of remission [68, 70]. These variables serve as pragmatic markers of disease severity. Patients requiring insulin or multiple agents are less likely to achieve remission, not because treatment itself impairs reversibility, but because pharmacologic burden often reflects advanced β-cell dysfunction and reduced metabolic reserve [68, 69]. Magnitude of Weight Loss The extent of weight loss is the most important modifiable predictor of remission. Evidence from DiRECT showed a strong dose-response relationship, with remission rates rising sharply as weight loss increased [70]. Similar findings have been reported in surgical cohorts, where greater postoperative weight loss is consistently associated with higher remission rates and lower risk of relapse [71]. This is likely consistent with greater reduction in hepatic and pancreatic fat, with corresponding improvements in insulin sensitivity and β-cell function [68, 72].

β-Cell Reserve Preserved β-cell function is a fundamental requirement for durable remission. Higher fasting or stimulated C-peptide levels and greater insulin secretory capacity independently predict remission following both lifestyle and surgical interventions [68, 72, 73] Mechanistically, interventions can reduce metabolic stress, but cannot fully restore normoglycemia in the absence of sufficient residual β-cell reserve [39].

## Challenges and Limitations

### Relapse After Remission

Despite high initial remission rates, particularly following bariatric surgery, relapse is common and represents a main limitation of current remission-oriented strategies [74]. Long-term data demonstrate a progressive decline in remission over time, with a substantial proportion of patients redeveloping hyperglycemia within 5-10 years [56, 74, 75]. Relapse is not random but is explained by underlying disease biology. Longer pre-intervention diabetes duration, greater baseline treatment burden, and inadequate or unsustained weight loss are consistently associated with loss of remission [64]. These patterns reinforce the concept that remission is biologically constrained, rather than uniformly achievable or permanent. Relapse should not be equated with treatment failure. Even after recurrence of hyperglycemia, patients typically maintain improved glycemic control, reduced medication requirements, and lower cardiometabolic risk compared with pre-intervention states [74]. This supports framing remission as part of a broader process of disease modification, rather than a binary cure [54, 69].

### Limitations of the Evidence Base

Despite substantial progress, important gaps remain in the evidence. Randomized controlled trials with follow-up beyond five years are limited, and those that exist often involve relatively small sample sizes and attrition bias. As a result, long-term durability of remission remains incompletely characterized [12]. Heterogeneity in remission definitions across studies further complicates interpretation. Differences in glycemic thresholds, duration requirements, and medication use criteria limit comparability and may lead to overestimation of durable remission [2, 47]. In addition, many long-term cohorts include outdated surgical procedures or techniques, reducing generalizability to current practice, particularly with respect to sleeve gastrectomy and modern Roux-en-Y approaches [44, 76, 77].

### Prediction and Uncertainty

Although several predictive models (e.g., DiaRem, ABCD) have been developed, their performance is modest and declines over longer time horizons [78]. Emerging evidence suggests that dynamic factors particularly early weight loss and short-term glycemic response may outperform static baseline models in predicting long-term outcomes [49].

### Safety, Cost, and Accessibility

Bariatric surgery, while highly effective, is resource-intensive and not universally accessible. Cost, healthcare infrastructure, and referral pathways limit its availability, particularly in low-resource settings and among socioeconomically disadvantaged populations [50]. Although perioperative risks are relatively low, long-term complications including micronutrient deficiencies, anemia, bone disease, and gastrointestinal disorders require lifelong monitoring [79]. More extensive procedures offer greater metabolic efficacy but at the expense of increased adverse events [80]. These considerations underscore a fundamental tension between efficacy and accessibility, which constrains the population-level impact of surgical interventions [49].

### Generalizability and Equity

The populations most likely to achieve remission are individuals with shorter diabetes duration lower HbA1c, and preserved β-cell function are often overrepresented in clinical trials [65]. Consequently, reported remission rates may reflect best-case scenarios rather than real-world populations [81]. Furthermore, many studies exclude individuals with psychiatric comorbidity, complex medical conditions, or socioeconomic barriers, limiting external validity and raising concerns about equity [81, 82]. Addressing these gaps will be essential to ensure that remission-oriented care is not only effective but also equitably delivered [82].

## Future Directions and Clinical Implications

The evolving evidence base for T2DM disease reversal necessitates a shift from a binary framework of “remission versus non-remission” toward a more nuanced model centerd on disease modification, durability, and long-term metabolic health [5]. Future research and clinical practice should prioritize strategies that extend beyond short-term glycemic targets to sustained physiological improvement [67].

### Refining Patient Selection and Stratification

A primary priority is improving the identification of patients most likely to achieve long-term durability of remission [67]. Current prediction models provide only moderate accuracy and are largely based on static clinical variables. Emerging evidence suggests that dynamic markers, particularly early weight loss and short-term glycemic response may better predict long-term outcomes [67, 77]. Integration of clinical, biochemical, and emerging omics-based markers including C-peptide, metabolomics, and gut microbiome profiles offers a promising avenue for more precise, individualised prediction [5]. Such approaches may enable a transition toward precision metabolic medicine, aligning interventions with underlying disease biology [1].

### Optimising Timing of Intervention

The consistent association between shorter diabetes duration and higher remission rates supports earlier, more proactive intervention. Current treatment pathways often delay escalation to intensive or surgical approaches until late disease stages, when reversibility is limited [57]. Reframing T2DM as a potentially reversible condition raises important questions regarding the optimal timing of intervention, including whether bariatric surgery should be considered earlier in the disease course for selected patients. Addressing this will require prospective trials directly comparing early versus delayed intervention strategies [65].

### Integrating Therapeutic Approaches

Rather than viewing lifestyle intervention and bariatric surgery as competing strategies, future care models should integrate these approaches within a continuum of metabolic care. Pre- and post-surgical lifestyle optimization, combined with emerging pharmacotherapies, may enhance both remission rates and durability [13, 62]. The increasing availability of highly effective anti-obesity and incretin-based therapies further complicates this landscape, raising important questions regarding sequencing, combination strategies, and long-term comparative effectiveness [51, 83, 84]. Long-term data beyond 10 years remain limited, with much of the evidence derived from selected cohorts and evolving surgical techniques, restricting generalizability [72, 85]. Major uncertainties include the durability of remission, relapse trajectories, and long-term complications. Increasing emphasis is placed on longitudinal outcomes, including sustained glycemic control, weight maintenance, and patient-centerd measures such as quality of life [47, 57, 83]. Real-world evidence from registries and routine clinical practice is therefore essential to complement randomized trials and provide a more accurate assessment of long-term effectiveness and safety [26, 71].

### Equity, Access, and Health Systems

Despite strong evidence of efficacy, bariatric surgery remains underutilized, particularly among populations with the highest burden of T2DM. Barriers include limited access, inconsistent referral pathways, and health system constraints [51]. Addressing these gaps requires integration of surgical options into standard diabetes care, improved referral processes, policy-level support for funding, and multidisciplinary management. Without these changes, remission-oriented strategies risk exacerbating existing health inequalities rather than reducing them [65, 86].

## Conclusion

Remission of T2DM is an achievable and clinically meaningful outcome, challenging the long-standing paradigm of inevitable disease progression. Substantial weight loss remains the dominant determinant of remission, acting through reduction of ectopic fat, restoration of insulin sensitivity, and partial recovery of β-cell function. While lifestyle interventions can induce remission, their long-term effectiveness is limited by challenges in sustaining weight loss. In contrast, bariatric surgery provides the most effective and durable pathway to remission, combining substantial weight loss with weight-independent metabolic effects. However, remission is not universal or permanent. Relapse is common and reflects underlying disease biology, reinforcing that T2DM is a potentially reversible but biologically constrained condition.

## Conflict of Interest

The authors declare that there are no conflicts of interest regarding the publication of this study.

## AI Disclosure Statement

During the preparation of this manuscript, the authors used ChatGPT for language editing, grammar improvement, and reference management support. After its use, the authors thoroughly reviewed, verified, and revised all AI-assisted content to ensure accuracy and originality. The authors take full responsibility for the integrity and final content of the published article.
